# Altered Corticobrainstem Connectivity during Spontaneous Fluctuations in Pain Intensity in Painful Trigeminal Neuropathy

**DOI:** 10.1523/ENEURO.0522-23.2024

**Published:** 2024-07-23

**Authors:** Noemi Meylakh, Lewis S. Crawford, Emily P. Mills, Vaughan G. Macefield, E. Russell Vickers, Paul M. Macey, Kevin A. Keay, Luke A. Henderson

**Affiliations:** ^1^School of Medical Sciences (Neuroscience), Brain and Mind Centre, University of Sydney, Sydney, New South Wales 2050, Australia; ^2^Department of Neuroscience, Monash University, Melbourne, Victoria 3800, Australia; ^3^UCLA School of Nursing and Brain Research Institute, University of California, Los Angeles, California 90095

**Keywords:** chronic pain, cortex, fMRI, functional connectivity, PAG, variability

## Abstract

Chronic neuropathic pain can result from nervous system injury and can persist in the absence of external stimuli. Although ongoing pain characterizes the disorder, in many individuals, the intensity of this ongoing pain fluctuates dramatically. Previously, it was identified that functional magnetic resonance imaging signal covariations between the midbrain periaqueductal gray (PAG) matter, rostral ventromedial medulla (RVM), and spinal trigeminal nucleus are associated with moment-to-moment fluctuations in pain intensity in individuals with painful trigeminal neuropathy (PTN). Since this brainstem circuit is modulated by higher brain input, we sought to determine which cortical sites might be influencing this brainstem network during spontaneous fluctuations in pain intensity. Over 12 min, we recorded the ongoing pain intensity in 24 PTN participants and classified them as fluctuating (*n* = 13) or stable (*n* = 11). Using a PAG seed, we identified connections between the PAG and emotional-affective sites such as the hippocampal and posterior cingulate cortices, the sensory-discriminative posterior insula, and cognitive-affective sites such as the dorsolateral prefrontal (dlPFC) and subgenual anterior cingulate cortices that were altered dependent on spontaneous high and low pain intensity. Additionally, sliding-window functional connectivity analysis revealed that the dlPFC–PAG connection anticorrelated with perceived pain intensity over the entire 12 min period. These findings reveal cortical systems underlying moment-to-moment changes in perceived pain in PTN, which likely cause dysregulation in the brainstem circuits previously identified, and consequently alter the appraisal of pain across time.

## Significance Statement

While the intensity of an individual's chronic pain is often measured at a specific point in time, it is known that in a large proportion of individuals, pain intensity fluctuates dramatically from moment-to-moment. In individuals with chronic neuropathic pain, we found that these spontaneous pain intensity fluctuations are associated with neural function fluctuations, specifically of function reflected as neural connectivity between brainstem pain-modulatory circuits and cortical regions, including the dorsolateral prefrontal (dlPFC) and cingulate cortices. These findings raise the possibility that modulating brain regions such as the dlPFC in individuals with fluctuating chronic pain may provide an avenue for analgesic treatment.

## Introduction

The perception of pain is vital to survival, warning us of danger and motivating us to remove ourselves from it. As such, pain is adaptive. However, if pain persists beyond the period of normal healing and extends for longer than 3 months, it is considered chronic in nature, maladaptive, and pathological. Chronic pain that results from the damage to the nervous system is called neuropathic pain and can occur in the absence of any external stimulus. In any one individual, the ongoing intensity of their pain can fluctuate between days, hours, or even from moment-to-moment. While the precise biological mechanisms underlying perceived pain intensity fluctuations in chronic pain patients are unclear, it is well known that the brainstem contains multiple circuits capable of modulating the intensity of perceived pain by modulating noxious information at the level of the dorsal horn and spinal trigeminal nucleus (SpV; Youssef et al., 2016; L. S. Crawford et al., 2022; Oliva et al., 2022).

Previous work has demonstrated the role of these circuits in chronic neuropathic pain by establishing that signal coupling between the midbrain periaqueductal gray (PAG) matter, rostral ventromedial medulla (RVM), and SpV was associated with perceived spontaneous pain intensity fluctuations in PTN patients (Mills et al., 2020). While this circuit is a well-established brainstem pain-modulatory network, whether changes in the connectivity of this circuit alone underpin spontaneous changes in pain intensity, or whether the connectivity of this brainstem circuit is also modulated by descending inputs from higher brain centers remains unknown. It is well known that the PAG receives descending modulatory influences from higher brain centers including areas such as the dorsolateral prefrontal cortex (dlPFC), anterior cingulate cortex (ACC), hippocampus, amygdala, and hypothalamus (Ongür et al., 1998; Eippert et al., 2009; Sevel et al., 2015).

These connections appear to drive changes in pain intensity in situations such as placebo and offset analgesia and nocebo hyperalgesia (L. S. Crawford et al., 2023a,b). Indeed, it was previously shown that signal covariation between these regions and the PAG are critical in determining whether an individual mounts a placebo analgesia (L. S. Crawford et al., 2023a). Furthermore, it has also been shown that a connection between the dlPFC and PAG drives pain intensity variability during repeated noxious stimuli of the same intensity in healthy participants (L. Crawford et al., 2023; L. S. Crawford et al., 2023b). Given these findings, it is likely that descending influences from higher brain regions also determine whether an individual displays spontaneous changes in ongoing pain intensity.

The aim of this investigation was to determine if signal covariations between higher cortical regions and the PAG are associated with moment-to-moment fluctuations in pain intensity in individuals with painful trigeminal neuropathy (PTN). We hypothesized that higher brain regions with well-established pain–modulatory ability such as the dlPFC, ACC, hippocampus, and amygdala would display increased functional connectivity with the PAG during periods of high relative to low pain intensity and would also display moment-to-moment functional connectivity changes with the PAG that were associated with fluctuations in perceived pain intensity.

## Materials and Methods

### Participants and pain measurements

Twenty-four PTN participants were recruited for the study (eight males; mean ± SEM age, 47.3 ± 2.9 years; range, 25–78 years). Diagnosis of the PTN presence was made in accordance with the Liverpool criteria (Nurmikko and Eldridge, 2001) by a clinician in the research group (E.R.V.). Inclusion criteria required a primary pain complaint relating to PTN and a pain intensity on the day of scanning >0. Exclusion criteria included typical MRI contraindications (current pregnancy, metallic implants), as well as the presence of any additional chronic pain condition in addition to PTN. Written informed consent was obtained from each participant for all procedures, approved by the University of Sydney Human Research Ethics Committee and consistent with the Declaration of Helsinki. Data from 24 PTN participants was used in previous studies (Mills et al., 2018, 2020).

For the 7 d preceding the day of scanning, PTN participants recorded the intensity of their ongoing pain three times a day using a visual analog scale (VAS; 0, no pain; 10, worst pain imaginable). The average of these pain ratings was taken as a measure of “diary pain intensity.” Participants also described their pain distribution by shading areas of a standard anatomical template of the face and described the quality of their ongoing pain through the completion of McGill's pain questionnaire (Melzack, 1975).

### Scanning procedures

Magnetic resonance imaging (MRI) was recorded during this study using a 3 Tesla MRI scanner (Achieva, Philips Medical Systems) at [Neuroscience Research Australia (NeuRA) in Randwick, Sydney]. Participants lay supine on a scanner bed with their head positioned within a 32-channel transmit and receive head coil. Ear buds and over ear headphones were provided to participants to reduce scanner noise, and sponges were placed between these headphones and the head coil to limit head motion throughout the course of functional scanning. A high-resolution T1–weighted anatomical image of the entire brain was collected (288 axial slices; repetition time, 5,600 ms; raw voxel size, 0.87 × 0.87 × 0.87 mm thick; acquisition time, 3 min 45 s). Following this, a series of 360 gradient-echo echo–planar brain volumes with blood oxygen level dependent (BOLD) contrast were recorded (37 axial slices; repetition time, 2,000 ms; echo time, 30 ms; raw voxel size, 3.0 × 3.0 × 4.0 mm thick; acquisition time, 12 min). Throughout this 12 min functional MRI (fMRI) scan, participants were instructed to rate their ongoing pain experience using a computerized VAS (CoVAS, Medoc). The CoVAS was connected to a digital screen visible to participants while inside the scanner, allowing them to report their ongoing pain intensity in real time (0, no pain; 10, worst pain imaginable). Pain intensity values were saved at the conclusion of each scanning session and subsequently averaged into 2 s rating periods overlying each functional volume for future analysis.

### MRI and statistical analysis

Structural and functional image preprocessing was performed using Statistical Parametric Mapping version 12 within MATLAB version R2023a (Friston et al., 1994; Ashburner et al., 2021). fMRI images were first realigned, and movement parameters were inspected to ensure that no participant displayed excessive motion throughout the 12 min scan (>1 mm rotational or >0.5° radian translational motion). These movement parameters were then modeled and removed from the fMRI signal through a linear modeling of realignment parameters procedure (Macey et al., 2004). Cardiac (frequency band of 60–120 beats per minute + 1 harmonic) and respiratory (frequency band of 8–25 breaths per minute + 1 harmonic) noise was modeled and removed using the Dynamic Retrospective Filtering (DRIFTER) toolbox (Särkkä et al., 2012). Each participant's fMRI image series was then linearly detrended to remove the effects of global signal intensity changes and coregistered to their corresponding T1-weighted anatomical image. The T1-weighted image was spatially normalized to the Montreal Neurological Institute (MNI) template using the Manual Computational Anatomy toolbox (CAT12; Seiger et al., 2018), and these same normalization parameters were applied to the fMRI image series. This process resulted in fMRI images being resliced into 2 × 2 × 2 mm voxels. Finally, for the functional connectivity analyses, we applied a temporal high-pass filter with a cutoff frequency of 0.02 Hz to the smoothed fMRI images to remove the low-frequency signals that cannot be resolved using 50 s windows (Leonardi and Van De Ville, 2015). Given that our elected seed resided within the midbrain PAG, a small structure with a discrete functional colocalization, all connectivity analyses were first performed using unsmoothed fMRI images to preserve the integrity of seed signal and seed-to-voxel relationships. The resulting connectivity contrast maps were then spatially smoothed using a 6 mm full-width at half-maximum (FWHM) Gaussian filter.

Since the equal number of PTN participants presented with unilateral left- and right-sided pain, functional images were flipped along the midline so that each participants’ resulting contrast images represented chronic pain in the left side of the face. Given that ascending pain pathways are largely represented contralaterally (at least in the case of the thalamus, the primary somatosensory and posterior insular cortices), this methodological consideration meant that we could produce a clear representation of the cortical sites receiving input from or driving the activity to the unilateral (right) PAG seed identified in previous investigations (Mills et al., 2020).

### Pain ratings

Inspection of pain intensity ratings across the entire 12 min of fMRI scanning revealed a stratification within PTN participants, such that in some individuals ongoing pain fluctuated greatly, while in others, pain remained relatively stable. In 11 participants, pain remained stable with these participants reporting an average difference between minimum and maximum pain of 0.2 out of 10 on the VAS (stable group). In the remaining 13 participants, ongoing pain intensity varied considerably, and in these participants, we observed an average difference in minimum to maximum pain of 3.4 out of 10 on the VAS (fluctuating group). For fMRI analyses, in order to match perceived pain intensity with changes in BOLD signal intensity and connectivity, ongoing pain intensity ratings for each participant were shifted forward two volumes (4 s). This shift accounted for the approximate hemodynamic delay since fMRI measures the oxygenation demands in discrete brain regions that follow 3–5 s after the neuronal response (West et al., 2019).

### PAG functional connectivity

**Figure 1.**
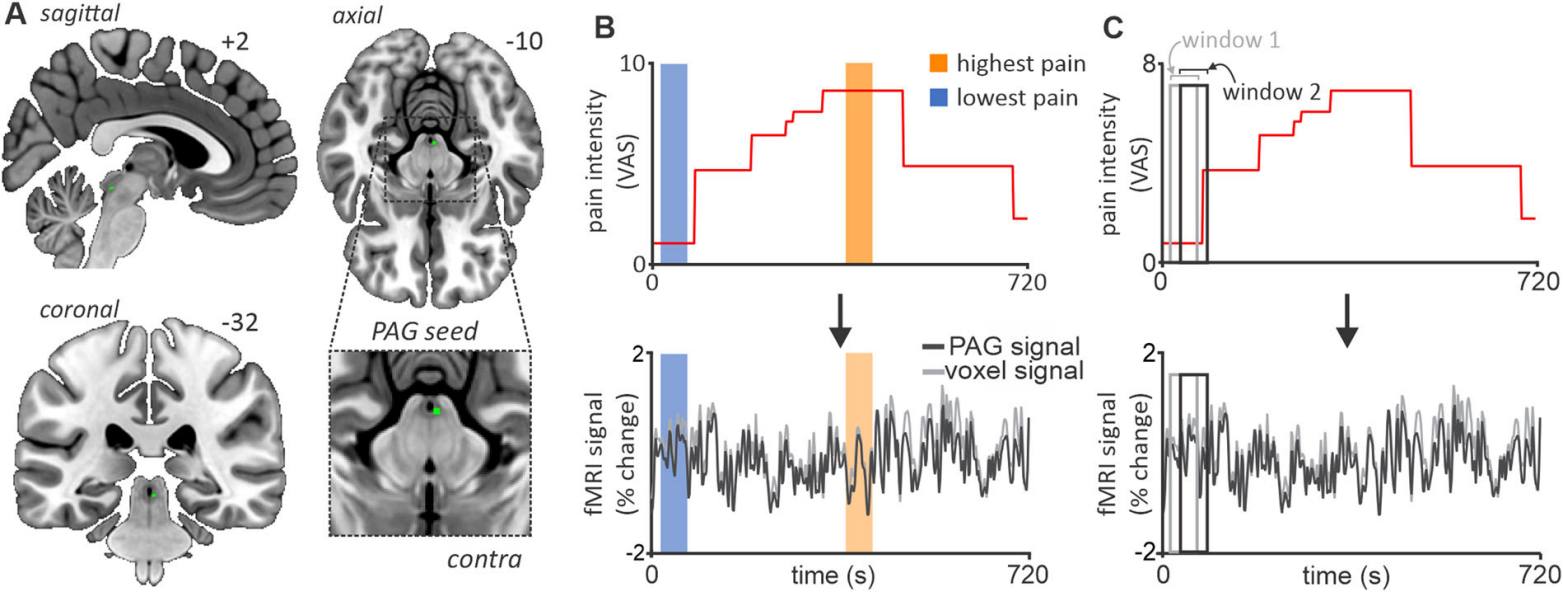
Midbrain PAG seed region, pain block, and sliding-window connectivity design. ***A***, With coordinates derived from a previous investigation utilizing this same dataset, a 1 mm sphere surrounding the PAG was generated and used as a seed region for subsequent connectivity analyses. ***B***, Visual representation of the bock analysis used to assess corticobrainstem functional connectivity during periods of spontaneously high versus spontaneously low pain. In this analysis, connectivity between the PAG seed and each cortical voxel was assessed within a 50 s period overlying participants lowest reported pain (blue shaded bar) against a 50 s period overlying participants’ highest reported pain (orange shaded bar). Pain intensity was assessed using a VAS from 0, no pain, to 10, worst pain imaginable. ***C***, In addition to the block design, a sliding-window analysis was performed where PAG connectivity was assessed during a 50 s period (Window 1 light gray box) which was then shifted forward by one volume (Window 2 dark gray box). This resulted in 336 contrast images representing 50 s mean PAG connectivity values across the entire 12 min scan.

Since a previous investigation (Mills et al., 2020) revealed the PAG, via relay with the RVM and SpV, to be an integral component of the descending brainstem circuitry responsible for spontaneous pain fluctuations in PTN (Mills et al., 2018), we were interested in determining the cortical inputs which first make contact with this brainstem circuitry to drive these effects. As such, we performed functional connectivity using the PAG cluster previously identified in brainstem-isolated space as a seed region for whole-brain voxel-by-voxel functional–connectivity analyses (Fig. 1*A*). In the present study, the PAG seed comprised eight contiguous voxels, four voxels at two rostrocaudal levels extending *z* = −8 to −10 in MNI space. The fMRI signal from each voxel within the seed was extracted and averaged to represent the mean PAG seed fMRI signal.

To study the functional cortical connections to the PAG, we performed separate functional connectivity analyses. We first calculated PAG connectivity within predefined time periods (blocks) corresponding to low and high pain intensities (Fig. 1*B*). Second, we discerned additional temporal information about these connections by using a sliding-window dynamic functional connectivity analysis to assess the relationship between PAG connectivity and pain intensity throughout the 12 min scan period (Fig. 1*C*).

### PAG high- versus low-pain block functional connectivity

First, we aimed to determine how PAG connectivity with cortical sites was different in PTN participants during periods of spontaneously high relative to spontaneously low perceived pain. To achieve this, for each of the 13 fluctuating pain participants, we calculated PAG connectivity in two separated 50 s (25 volumes) time periods (blocks)—one during the lowest perceived pain period across the 12 min fMRI scan and the other during the highest perceived pain (Fig. 1*B*). Importantly, to minimize any motion-related artefact relating to moving the CoVAS slider, we isolated blocks where pain remained relatively stable for an entire 50 s period and avoided any periods when a large (>1 cm) volume-to-volume movement along the CoVAS occurred. For each block, using each participant's fMRI images, we conducted first-level analyses with timeseries of PAG seed signal within the specified block attached as a condition, similar boxcar models traditionally used in task-related fMRI analyses. This first level resulted in the generation of two contrast images for each participant, one where voxels of greater beta value represented greater PAG connectivity during spontaneously low pain and the other during spontaneously high pain. These images were spatially smoothed with a 6 mm FWHM Gaussian filter. These contrast images reflecting connectivity map effects were entered into a second-level, paired, random-effect analysis of all 13 fluctuating pain participants. We set an initial threshold of *p* < 0.001 uncorrected for multiple comparisons, with a cluster defining a threshold of 10 contiguous voxels, and each resulting cluster was subject to small volume correction (svc) for multiple comparisons to reduce the likelihood of Type 1 errors.

Within each resulting cluster, we extracted PAG connectivity strength values for both lowest- and highest-pain blocks in each fluctuating pain participant and plotted mean (±SEM) functional connectivity strengths to provide a measure of connectivity direction (i.e., correlated or anticorrelated). These connectivity strengths were then entered into post hoc paired *t* tests to determine the direction and degree of significance in change of PAG connectivity between low- and high-pain blocks (Bonferroni-corrected for multiple comparisons). In addition, because the majority of participants with fluctuating pain showed not one single VAS movement but several throughout the scan, which corresponded to pain intensity values which were between the lowest and highest blocks, we further assessed whether the sites we identified in the initial paired analysis also displayed a “dose response” with PAG connectivity and perceived pain. That is, additional blocks were included where possible where PAG signal was extracted, and contrast maps were generated during 50 s periods of pain closer to the lowest reported intensity (middle-low) or closer to the highest reported intensity (middle-high). This process resulted in a maximum of four PAG connectivity blocks for each cluster that represented fluctuating pain during lowest (Block 1) to highest (Block 4) pain in each participant.

**Figure 2.**
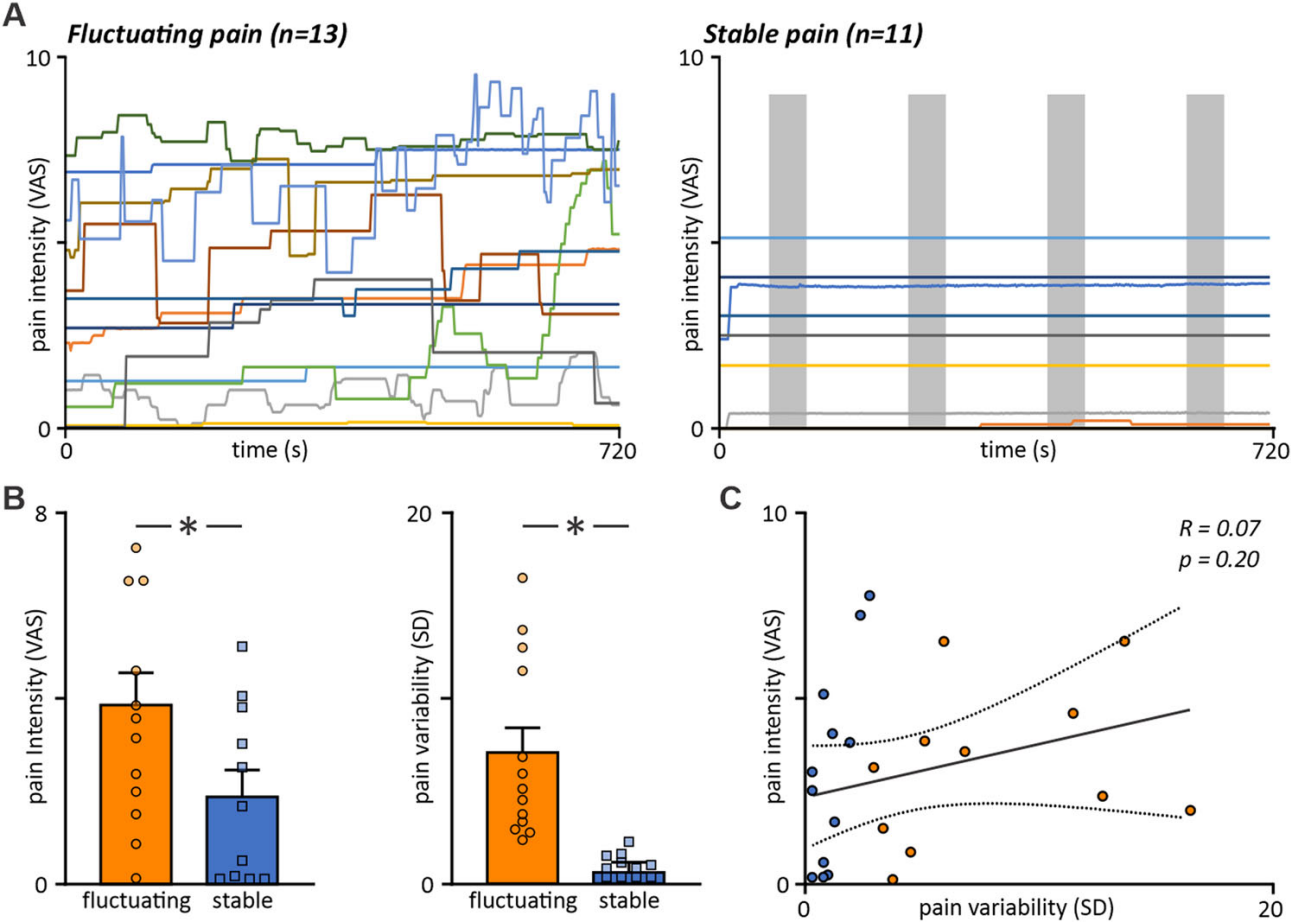
Ongoing pain intensity ratings and pain intensity variability in fluctuating (*n* = 13) and stable (*n* = 11) pain groups. ***A***, Trace of VAS responses recorded throughout the entire 12 min fMRI scan series. All participants fit the diagnostic criteria for trigeminal neuropathic pain; however, note that multiple spontaneous shifts in perceived pain are observed in only our fluctuating pain group (left plot). ***B***, Mean pain intensity and pain rating variability in fluctuating (orange bars) and stable (blue bars) pain groups across the 12 min scan. Fluctuating pain participants exhibited significantly higher mean intensity and variability of pain than stable participants. ***C***, Linear regression analysis comparing participant mean pain intensity and variability throughout the 12 min scan period. No significant interaction between these variables was observed despite variable participants demonstrating both greater intensity and variability than stable pain participants.

To assess PAG connectivity over time in the 11 participants who displayed stable pain over the 12 min scanning period, we calculated PAG connectivity in four equally spaced 50 s blocks (Fig. 2*A*). For each significant cluster identified in our fluctuating pain participant low- versus high-pain block analysis, we extracted and plotted the mean PAG connectivity for all four blocks within the stable pain participants.

### PAG sliding-window functional connectivity

Since it is likely that cortical structures engage pain-modulatory circuits of the brainstem not only when pain is spontaneously high or low but also across a continuum, we determined if there was an ongoing relationship between fluctuating and spontaneous pain intensity and PAG connectivity strength. Eleven of the 13 fluctuating pain participants displayed multiple (>3) pain intensity changes throughout the 12 min scanning period. Using the Dynamic BC toolbox (Liao et al., 2014), in each of these 11 participants, fMRI image sets were divided into 50 s (25 volume) windows with a repetition time of 2 s, resulting in 336 sliding windows (Fig. 1*C*). For each of these windows, a Pearson's correlation coefficient was calculated between the PAG seed and each cortical voxel, resulting in a series of 336 dynamic PAG connectivity maps per participant. After undergoing spatial smoothing (6 mm FWHM), these dynamic PAG connectivity maps were entered into a first-level linear regression analysis in which PAG dynamic functional connectivity was compared with 336 corresponding sliding-window pain intensity ratings. This resulted in a single contrast image where more positive beta values represented voxels where ongoing coupling with the PAG closely followed spontaneous pain fluctuations, that is, when voxel–PAG connectivity was higher, so too was perceived pain and vice versa for low pain.

These contrast images were then entered into a second-level one–sample random effects analysis to determine cortical sites in which dynamic PAG functional connectivity significantly correlated with perceived pain in each of the 11 fluctuating pain participants. We set an initial threshold of *p* < 0.001 uncorrected for multiple comparisons, with a cluster defining threshold of 10 contiguous voxels, and each resulting cluster was subject to svc to reduce the likelihood of Type 1 errors.

## Results

### PTN participant characteristics

Individual PTN participant characteristics are displayed in Table 1. Mean (±SEM) pain in the 7 d preceding the MRI session (diary pain) was 3.8 ± 0.4 out of 10 with a mean PTN pain duration of 5.2 ± 1.3 years. All 24 PTN participants recorded current pain on the day of scanning, frequently selecting words to describe their pain such as throbbing (39%), sharp (26%), and exhausting (37%). Across the 12 min of scanning, 13 participants reported multiple fluctuations in pain intensity, with 11 participants reporting relatively stable pain intensity—altering VAS slider position a maximum of once within the entire 12 min period (Fig. 2*A*). Variable and consistent PTN participants did not differ significantly in terms of age (mean* ± *SEM years, variable, 43.7 ± 4.0; consistent, 51.5 ± 4.0; *p* = 0.20), pain diary intensity (mean* ± *SEM pain, variable, 4.3 ± 0.6; consistent, 3.1 ± 0.4; *p* = 0.15), or years lived with PTN pain (mean ± SEM years, variable, 4.6 ± 1.8; consistent, 5.9 ± 1.8; *p* = 0.66) (two-sample *t* test). Furthermore, the previous 7 d mean pain intensities were not predictive of pain during the scan which is revealed by the lack of correlation (*R* = 0.22) and no statistical significance (*p* = 0.33) between these measures. Of the 13 variable PTN participants, 6 reported predominantly left-sided pain and 7 predominantly right-sided pain. In the 11 consistent PTN participants, 3 reported predominantly right-sided pain, 5 left-sided pain, and 3 reported bilateral pain.

**Table 1.** PTN participant classification

	Age (years)	Sex	Side of pain	Diary pain intensity (VAS)	Diary pain variability (SD)	Pain duration (months)	Current medication use
1	29	F	Left	5.3	1.5	49	-
2	45	M	Left	3.1	0.4	21	-
3	35	M	Right	1.5	0.7	96	PEA
4	78	F	Left	1.9	0.7	314	Paracetamol, Ostelin, Pristiq, Somac
5	47	F	Right	2.1	0.4	13	PEA
6	34	M	Right	3.7	1.7	10	PEA, Mersyndol
7	66	M	Left	3.9	0.6	48	-
8	44	F	Right	-	-	5	Valium, Panadeine Forte
9	47	F	Right	4.1	1.6	7	-
10	44	F	Left	8.1	0.4	75	PEA
11	46	F	Left	7.5	2.5	27	Endep
12	28	F	Right	5.8	0.3	52	-
13	25	M	Right	4.5	1.7	8	Panadeine Forte
14	69	F	Right	3.9	0.4	259	Endep, Lyrica, Oroxine, Crestor, Effexor, folic acid, prednisone
15	45	F	Bilateral	2.8	2.8	24	Duloxetine
16	66	M	Right	1.8	0.7	81	Endep
17	36	F	Left	1.9	0.6	37	PEA
18	40	F	Left	5.8	0.3	33	-
19	58	F	Left	4.0	0.4	11	Namipril, Zoloft, Panadol, PEA
20	35	M	Left	4.2	0.7	60	-
21	67	M	Bilateral	2.0	0.9	96	Endep
22	51	F	Left	1.5	0.3	13	Mobic, PEA
23	35	F	Right	3.4	1.0	15	Amitriptyline
24	64	F	Bilateral	-	-	144	-

Participants 1–13 were placed in the fluctuating pain group, and Participants 14–23 were placed in the stable pain group. PEA, palmitoylethanolamide; VAS, visual analog scale; SD, standard deviation.

During the 12 min fMRI scan, however, compared with PTN participants with consistent pain ratings, those with variable ratings demonstrated significantly greater ongoing pain intensity ratings (mean* ± *SEM VAS pain, variable, 3.9 ± 0.7; consistent, 1.9 ± 0.6; *p* < 0.05), as well as pain intensity variability (mean* ± *SEM VAS variability, variable, 7.1 ± 1.3; consistent, 0.4 ± 0.2; *p* < 0.05; two-sample *t* tests; Fig. 2*B*). Despite this, after performing linear regression analyses in all 24 PTN participants, no significant interaction was observed between ongoing pain intensity and variability across the entire 12 min scanning period (*R* = 0.27; *p* = 0.20; Fig. 2*C*). For the 13 fluctuating PTN participants, mean (±SEM) VAS pain intensity during the four pain blocks used for subsequent connectivity analyses were Block 1 (lowest pain; *n* = 13) 2.9 ± 0.7, Block 2 (*n* = 11) 3.7 ± 0.4, Block 3 (*n* = 10) 4.0 ± 0.8, Block 4 (highest pain; *n* = 13) 5.0 ± 0.7.

### PAG high- versus low-pain block functional connectivity

**Figure 3.**
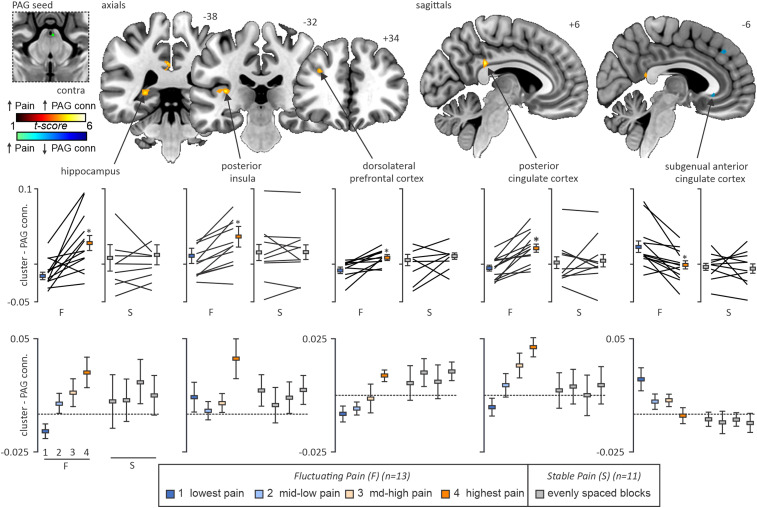
Significant differences in PAG connectivity strength during periods of high versus low pain in fluctuating pain participants (*n* = 13). Across the 12 min scanning window, the 25 volumes where participants rated their pain as the most intense were entered into a second-level, paired design with the 25 volumes where participants rated their pain the least intense. The top panel shows significant cortical clusters with greater midbrain PAG connectivity during high versus low pain (red color scale) and vice versa (blue color scale). The slice locations in MNI space are shown at the top right of each slice. The middle panel shows plots of mean (±SEM) PAG connectivity extracted from the above clusters and compared between fluctuating (high- and low-blocks) and stable (evenly dispersed blocks) participants. Note that the magnitude of PAG connectivity change in these sites only differs in those participants where pain fluctuates. Within the bottom panel is a post hoc analysis of the dose-dependent response within these clusters, as across the scan course our fluctuating pain group exhibited periods of perceived pain between the lowest and highest reported blocks. Note that in the hippocampus, dlPFC, and PCC, PAG connectivity appears to particularly track with the magnitude of pain only in those participants displaying fluctuating pain. Please see Extended Data Figure 3-1.

Our voxel-by-voxel analysis comparing PAG functional connectivity during periods of spontaneously high versus low pain in our fluctuating PTN participants revealed several cortical regions displaying pain intensity-related changes. Significantly greater PAG connectivity strength during high relative to low pain was identified in the ipsilateral posterior insular cortex (mean ± SEM PAG connectivity beta value lowest pain vs highest pain, 0.011 ± 0.009 vs 0.037 ± 0.013), ipsilateral hippocampus (−0.011 ± 0.005 vs 0.028 ± 0.010), ipsilateral dlPFC (−0.007 ± 0.004 vs 0.007 ± 0.003), and the posterior cingulate cortex (PCC; −0.005 ± 0.004 vs 0.021 ± 0.006). The inverse contrast revealed a single cluster satisfying correction in the subgenual ACC (sgACC) which displayed significantly greater PAG connectivity during periods of spontaneously low compared with high pain (0.023 ± 0.008 vs −0.001 ± 0.005; Fig. 3; Table 2; paired *t* tests; all *p* < 0.001 uncorrected for multiple comparisons, small volume corrected). No significant linear relationship was observed between PAG connectivity within each of these clusters during low- or high-pain periods and either mean pain diary intensity or variability in the 7 d preceding the scan (Table 1).

10.1523/ENEURO.0522-23.2024.f3-1 Figure 3-1 Mean connectivity and two-factor ANOVA interaction significance within cortical regions identified as altering in connectivity with the midbrain periaqueductal gray between low and high pain periods in fluctuating pain PTN participants. Note that only fluctuating PTN pain participants that displayed pain intensity ratings in all four pain blocks were included in this post-hoc analysis. *Ipsi = ipsilateral, PI = posterior insula, PCC = posterior cingulate cortex, dlPFC = dorsolateral prefrontal cortex, sgACC = subgenual anterior cingulate cortex*. Download Figure 3-1, DOCX file.

**Table 2.** MNI coordinates, cluster sizes, and significance values for clusters showing midbrain PAG connectivity differences during spontaneous low- versus high-pain intensity blocks in fluctuating pain participants (*n* = 13)

Region	MNI coordinate			Cluster size (kE)	*t* value	*z* value
	*X*	*Y*	*Z*			
Highest > lowest pain						
Ipsilateral posterior insula	−36	−32	8	21	6.08	4.03
PCC	−6	−36	30	18	5.16	3.68
Ipsilateral hippocampus	−52	0	2	11	3.86	3.05
Ipsilateral dlPFC	−20	34	28	11	3.77	3.00
Highest < lowest pain						
sgACC	6	−28	−6	12	3.86	3.05

For each of these clusters, PAG connectivity strength was also extracted from additional blocks of increasing pain throughout the scan for a maximum of four graded pain blocks (Block 2, moderate-low pain; Block 3, moderate-high pain). Within the ipsilateral hippocampus (mean ± SEM PAG connectivity beta value, Blocks 2, 3: 0.007 ± 0.007, 0.014 ± 0.009), dlPFC (0.005 ± 0.005, 0.013 ± 0.008), and PCC (0.005 ± 0.005, 0.013 ± 0.008) clusters, we observed a graded response of PAG connectivity change such that PAG connectivity closely followed spontaneous pain as it moved higher/lower throughout the scan. Conversely, clusters within the ipsilateral posterior insula and sgACC did not clearly show this graded response effect. Visual inspection of connectivity patterns within these clusters showed a strikingly similar effect between each of our 13 fluctuating PTN participants, with 12 of the 13 participants showing increases between low- and high-pain periods in PAG–hippocampus, PAG–posterior insula, and PAG–dlPFC. Likewise, 12 of the 13 participants showed decreases between low- and high-pain periods in PAG–sgACC connectivity.

Within each of these five clusters, measures of PAG connectivity in stable pain PTN participants were extracted within four evenly distributed scan windows as described previously. In no single cluster did we identify changes in PAG connectivity throughout the scan duration. That is, within the 11 stable PTN participants, PAG connectivity with the ipsilateral posterior insula (mean ± SEM PAG connectivity beta value, Blocks 1, 2, 3, 4: 0.016 ± 0.010, 0.006 ± 0.012, 0.011 ± 0.010, 0.016 ± 0.010), the PCC (0.002 ± 0.008, 0.004 ± 0.008, 0.001 ± 0.009, 0.005 ± 0.008), the ipsilateral hippocampus (0.008 ± 0.018, 0.009 ± 0.014, 0.021 ± 0.015, 0.013 ± 0.013), the ipsilateral dlPFC (0.005 ± 0.008, 0.010 ± 0.006, 0.006 ± 0.007, 0.010 ± 0.004), nor the sgACC (−0.003 ± 0.005, 0.005 ± 0.007, 0.004 ± 0.005, 0.006 ± 0.006) was significantly altered between any of the four pain blocks (one-way ANOVA, *p* > 0.05). Moreover, post hoc two-factor repeated ANOVA analyses revealed a significant interaction between group assignment (i.e., “stable” or “fluctuating” PTN pain) and PAG connectivity strength with each of these five regions between each increasing intensity pain block (Extended Data Fig. 3-1).

### PAG sliding-window connectivity

**Figure 4.**
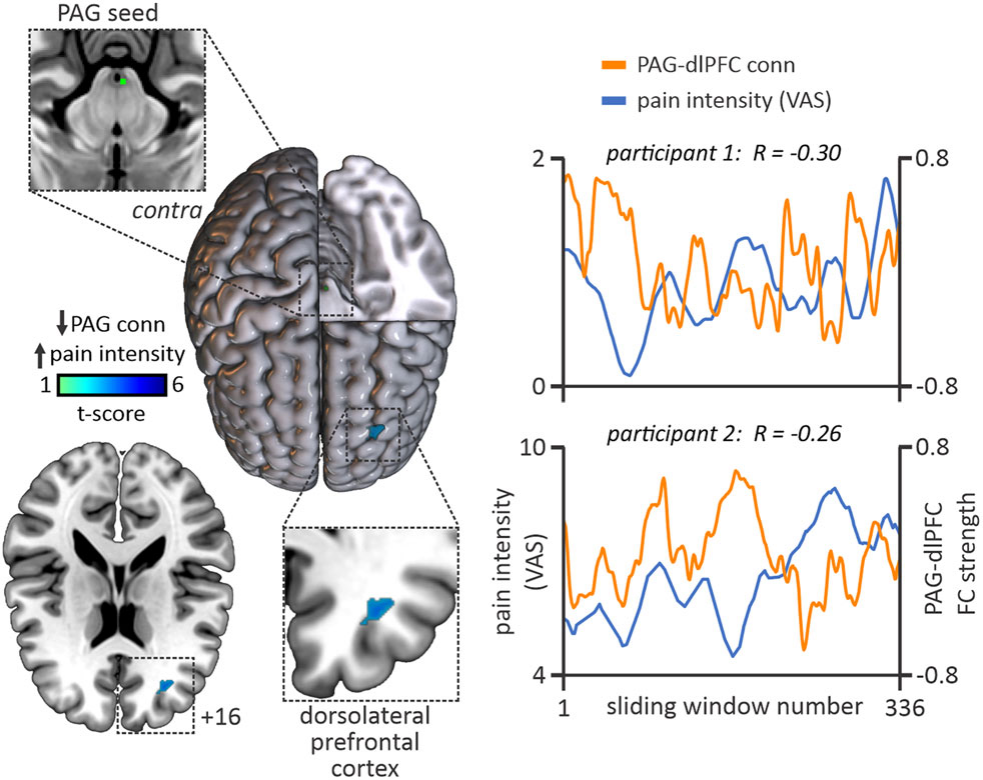
Cortical region demonstrating ongoing PAG connectivity change relating to fluctuations in pain intensity perceived across the 12 min scan period. The left overlay shows firstly the midbrain PAG seed (top box) sectioned within a render also showing a significant cluster in the contralateral (to side of pain) dlPFC where PAG connectivity was anticorrelated with perceived pain throughout the entire scanning period in fluctuating pain patients. The right panel provides visual examples of two fluctuating pain PTN participants where PAG–dlPFC connectivity (orange line) was continuously anticorrelated with perceived pain (blue line) over the 12 min scanning window. FC, functional connectivity; VAS, visual analog scale; dlPFC, dorsolateral prefrontal cortex.

Dynamic functional connectivity analysis revealed that in our group of fluctuating PTN participants, PAG dynamic functional connectivity was inversely correlated with sliding-window pain intensity over the entire scan in a region of the contralateral (to side of predominant pain) dlPFC (Fig. 4; Table 3). We extracted and temporally smoothed the PAG dynamic connectivity values within the dlPFC cluster, identifying a significant inverse relationship between ongoing pain intensity ratings and PAG–dlPFC connectivity in 9 of the 11 participants (*p* < 0.05). That is, as pain was spontaneously high throughout the 12 min scanning period, PAG–dlPFC connectivity was more anticorrelated and vice versa. Two individual participant plots of pain versus PAG–dlPFC connectivity are shown in Figure 4. PAG dynamic connectivity positively correlated with pain intensity in no cortical region.

**Table 3.** MNI coordinates, cluster sizes, and significance values for clusters showing correlations pain intensity and PAG connectivity across the entire 12 min scan length in fluctuating pain participants (*n* = 13)

Region	MNI coordinate			Cluster size (kE)	*t* value	*z* value
	*X*	*Y*	*Z*			
Negative correlation						
Contralateral dlPFC	32	40	16	21	4.55	3.27

## Discussion

The results of this study highlight that in individuals with chronic neuropathic pain, fluctuations in ongoing pain intensity are associated with altered communication between cortical areas with known roles in pain processing and modulation and pain-modulatory circuitry of the brainstem. That is, when pain was spontaneously high, we observed higher PAG coupling with the hippocampus, as well as the posterior insula, dlPFC, and PCC compared with periods where pain was spontaneously low. Conversely, when pain was spontaneously low, greater PAG coupling with the sgACC occurred compared with when pain was spontaneously high. These coupling changes were isolated to the fluctuating PTN participant group, with no overall changes in PAG coupling within these sites in PTN participants where pain remained stable. Additionally, our sliding-window analysis revealed that throughout the entire 12 min scanning window, PAG–dlPFC coupling was negatively associated with ongoing pain intensity ratings. That is, the higher the pain, the lower the PAG–dlPFC coupling and vice versa. These data show that there are moment-to moment changes in cortical signaling to, or reception of signals from, brainstem pain-modulatory circuits residing in the midbrain PAG, and these likely contribute to fluctuations in spontaneous pain intensity in individuals suffering chronic neuropathic pain.

It is well established that the PAG is capable of altering the perception of pain either by descending input to the spinal cord dorsal horn (for the body) or SpV (for the face) via relay with the RVM or by ascending inputs to the cortical sites responsible for the pain percept (Zambreanu et al., 2005; Fairhurst et al., 2007; Heinricher et al., 2009; Ong et al., 2019). In pain-free individuals, the PAG–RVM descending pathway is believed finely balanced to manage nociceptive load, enabling analgesia when pain is unnecessary (such as when fighting or fleeing) or hyperalgesia to enable rest and healing behaviors. However, in individuals with chronic neuropathic pain, it has been suggested that this pathway is shifted, driven by cortical input, to promote a baseline pronociceptive state (Ossipov et al., 2014; Chen et al., 2018; Henderson and Keay, 2018). Indeed, it was previously established that this PAG–RVM circuitry was modulated in this group of PTN patients, with altered connectivity patterns between the PAG–RVM–SpV in those where pain fluctuated considerably over time. Here we establish the likely top–down cortical inputs which contact this network to drive pain intensity fluctuations.

It has been previously proposed that the dlPFC acts as a cortical interface between cognitive processing and pain modulation, performing this role by communicating with the PAG both directly and via the ACC (Eippert et al., 2009; Sevel et al., 2015). Directed connectivity analyses previously identified decreases in dlPFC–PAG coupling as predictive of greater placebo analgesia in a healthy population (Sevel et al., 2015), and noninvasive stimulation of the dlPFC can both attenuate and amplify endogenous pain-modulatory responses (Krummenacher et al., 2010; Tu et al., 2021). Moreover, stimulation of the dlPFC in chronic pain patients can enhance pain tolerance and alleviate pain symptoms (Graff-Guerrero et al., 2005; Leung et al., 2018). Previously, the function of the dlPFC was extended to be involved in moment-to-moment pain intensity changes in healthy humans, with the dlPFC demonstrating reduced activation, alongside altered coupling patterns with pain processing areas such as the posterior insula and primary somatosensory cortex, in individuals who express greater variation in perceived pain intensity during a series of identical noxious stimuli (L. Crawford et al., 2023). The results of the present investigation suggest that this dlPFC role is also operating in chronic pain patients—via descending input to the PAG—with both block and sliding-window connectivity analyses showing dlPFC–PAG connectivity is associated with moment-to-moment fluctuations in pain intensity. Additionally, top–down connections from the dlPFC to PAG are known to encode not only pain modulation but also threat responses to an individual's immediate environment. Wang and colleagues demonstrated the functional connectivity patterns of distinct PAG columns during threat processing, showing that more negative PAG–dlPFC coupling during high-threat relative to low-threat states (Wang et al., 2022). Given that greater pain, especially when inside the confined environment of an MRI scanner, likely imposes a high threat to body homeostasis, ongoing and updating signals from the dlPFC to the PAG could encode such a threat.

Similar to the dlPFC, we identified that coupling between the PAG and posterior insula increased with perceived pain intensity. The posterior insular appears to encode sensory components of pain and shows functional activation correlates with pain intensity in healthy humans (Wager et al., 2013). Uniquely, the posterior insular is one of the only cortical sites where direct stimulation can trigger pain and where cortical injury can cause the manifestation of neuropathic pain symptoms (Mazzola et al., 2012; Bergeron et al., 2021). Furthermore, viral tract tracing in nonhuman primates demonstrates the posterior insula as the primary target for projection neurons arising from the spinothalamic tract (Dum et al., 2009), consistent with a role in detecting and encoding the magnitude of incoming pain signals. Since our fluctuating group displayed significantly higher variability of ongoing pain and greater overall pain intensity, the posterior insula may code both overall pain intensity and modulate the PAG to alter moment-to-moment pain intensity fluctuations.

It has been proposed that brain regions that code the emotional-affective aspects of pain, such as the hippocampus and posterior cingulate, play a more central role in chronic pain than they do during acute pain (Garcia-Larrea and Peyron, 2013). In humans, the connection between the hippocampus and PAG is believed to encode the memory and retrieval of pain and threat responses (Mokhtari et al., 2019; Wang et al., 2022), and in experimental animals, chemogenic modulation of hippocampal function can suppress neuropathic pain behaviors via direct modulation of sensory processing sites (Xuhong et al., 2018). Furthermore, human chronic pain patients demonstrate increased ongoing activation in the PCC when compared with controls, and in fibromyalgia patients, this increased activation has shown to correlate with pain catastrophizing or negative affect toward pain symptoms (Hsieh et al., 1995; Lee et al., 2018). Our data indicate that spontaneous changes in pain intensity in PTN patients are also encoded in these emotional-affective cortical regions, which may play a direct role in modulating the intensity of ongoing pain.

The sgACC was the only cortical region in which we found PAG connectivity to decrease as pain intensity spontaneously increased. The sgACC was originally proposed by Vogt and colleagues to rarely encode acute pain responses and instead is integral for producing emotional responses to pain via direct autonomic projections to subcortical structures such as the central nucleus of the amygdala and the PAG (Vogt, 2005). These connections however have shown not only to encode emotional responses but the degree of pain-modulatory capacity in healthy humans. Specifically, in a large human cohort, Cheng and colleagues showed that greater sgACC and brainstem coupling reduced the intensity of temporal summation (Cheng et al., 2015). Similarly, in a longitudinal study, Bingel and colleagues found that activation of the sgACC related to the magnitude of habituation over time, suggesting a core role of this site in suppressing nociceptive responses through recruitment of antinociceptive circuits (Bingel et al., 2008). Our data not only support the role of the sgACC in inhibiting pain responses over time but further suggest that in PTN participants where pain fluctuates, the sgACC–PAG connection is inhibited to facilitate an increase in pain intensity.

### Limitations

This study comes with several caveats. Firstly, we reflected some participants’ images so that the left side of the brain corresponded to the side of “most pain.” While this approach was used to keep the pain side consistent, we appreciate that some higher brain regions display laterality and thus some information may have been diluted by this process. Secondly, since participants were required to operate the VAS manually by sliding their fingers along its extent, cortical activation of motor networks may have been induced that could alter our interpretation. To counter this, when conducting our pain block analysis, we isolated scan periods where pain remained relatively stable within the 25-volume period of interest. Additionally, only the dlPFC survived correction in the dynamic functional connectivity analysis, which is not likely to be involved in directing hand movements, and any motion effects would be averaged across the 50 s period comprising each of the 336 time windows. Thirdly, though our sample can detect a medium effect size with a power of 0.95, the relatively small sample size in both our fluctuating and stable pain groups could limit overall generalizability, although these sample sizes are similar to previous studies investigating ongoing pain fluctuations in chronic pain disorders (Baliki et al., 2006, 2011; Foss et al., 2006; Geha et al., 2007). Finally, the choice to employ sliding-window connectivity over a “static connectivity” measure using each scan volume independently is worth noting. While it is unclear whether averaging seed-to-voxel signal in short (50 s) periods measures similar processes observed across a total scan period, this technique has previously demonstrated utility in tying behavioral variables such as ongoing shifts in vigilance and respiration with physiological ones such as brain network dynamics (Chang et al., 2013; Thompson et al., 2013). Indeed, this investigation utilized a similar linear regression approach to Chang and colleagues, assessing whether PAG connectivity with the cortex tracks pain intensity over time.

## Conclusion

Overall, our data reveal that cortical sites interact with the brainstem circuits responsible for ongoing pain intensity fluctuations in patients with chronic orofacial neuropathic pain. While it is well known that areas like the dlPFC and ACC are involved in pain modulation in pain-free humans, we extend this idea and show that in individuals with ongoing pain, perceived intensity can fluctuate moment-to-moment and we show here such fluctuation is associated with descending analgesic circuitry function. Given that areas such as the dlPFC are accessible to manipulation through techniques such as transcranial direct current stimulation, transcranial alternating current stimulation (tACS), or repetitive transcranial magnetic stimulation, these findings may provide the platform for testing the analgesic ability of the dlPFC in chronic pain, particularly in those individuals that display variations in ongoing pain intensity. Indeed, we have recently shown that tACS of the dlPFC can engage subcortical circuitry involved in the generation of sympathetic outflow to the muscle and skin, likely via the PAG (Sesa-Ashton et al., 2022; Wong et al., 2023).

## Synthesis

Reviewing Editor: Jennifer Dulin, Texas A&M University Decisions are customarily a result of the Reviewing Editor and the peer reviewers coming together and discussing their recommendations until a consensus is reached. When revisions are invited, a fact-based synthesis statement explaining their decision and outlining what is needed to prepare a revision will be listed below. The following reviewer(s) agreed to reveal their identity: Jan Haaker.

Synthesis: This manuscript describes cortical systems associated with fluctuations in neuropathic pain in human subjects, and identifies connections between the periaqueductal gray and emotional-affective sites that were altered dependent on spontaneous self-reported high and low pain intensity. The manuscript reports novel findings that will be of broad interest to the pain community. However, some issues preclude publication in eNeuro at this time. Please see detailed point-by-point review below.

Reviewer's Comments:

Reviewer #1:

The manuscript provides an interesting and novel way to analyse pain processing by characterising hemodynamic changes during spontaneous pain fluctuation (in a subset of N=13). These analyses reveal interesting interactions of cortical regions with the PAG that may be related to the endogenous regulation of pain.

1) The resolution of the functional MRI scans is rather low (with 3x3x4mm resliced to 2x2x2mm) to study the PAG. Can the authors assure us that their resolution is sufficient to identify the regions of interest?

2) The comparison of PAG connectivity across four blocks showed altered connectivity in the fluctuating group but not in the group reporting stable pain (line 317 and following). While such a within-group comparison is interesting for descriptive purposes, the group comparison is lacking (i.e. detecting a significant effect in one group but not the other does not qualify as a statistical comparison: https://elifesciences.org/articles/48175). It would be interesting to test whether the variation in PAG connectivity in the fluctuating group differs from that in the stable group (e.g. within a two-factor ANOVA).

3) To address the limitation of the small sample size, the authors could perform a sensitivity analysis that would reveal the critical F-value (or effect size) that their sample is able to derive. This will allow the reader to interpret the different effect sizes of the results in the light of the power that this sample can derive.

4) The methods should state that the sample has already been published in another analysis.

5) Are the mean intensities or variations of the previous seven days predictive of a) pain intensities or variations during the scan day or predictive of PAG connectivity measures? Such an analysis might support the fact that it is indeed "spontaneous" fluctuations that drive the effect, rather than inter-individual differences or a history of pain fluctuations.

6) The group sizes of the "fluctuating" and "stable" group should inserted in the abstract " ..we recorded ongoing pain intensity in 24 PTN participants, and classified them as fluctuating or stable"
