# Supplementary material for: Altered Corticobrainstem Connectivity during Spontaneous Fluctuations in Pain Intensity in Painful Trigeminal Neuropathy
Source: eNeuro. 2024 Jul 23;11(7):ENEURO.0522-23.2024. doi: 10.1523/ENEURO.0522-23.2024 (PMC11277291; doi:10.1523/ENEURO.0522-23.2024)
Supplement: Figure 3-1 — Mean connectivity and two-factor ANOVA interaction significance within cortical regions identified as altering in connectivity with the midbrain periaqueductal gray between low and high pain periods in fluctuating pain PTN participants. Note that only fluctuating PTN pain participants that displayed pain intensity ratings in all four pain blocks were included in this post-hoc analysis. Ipsi = ipsilateral, PI = posterior insula, PCC = posterior cingulate cortex, dlPFC = dorsolateral prefrontal cortex, sgACC = subgenual anterior cingulate cortex. Download Figure 3-1, DOCX file. [file eneuro-11-ENEURO.0522-23.2024-s001.docx]

**Figure 3-1.** Mean connectivity and two-factor ANOVA interaction significance within cortical regions identified as altering in connectivity with the midbrain periaqueductal gray between low and high pain periods in fluctuating pain PTN participants. Note that only fluctuating PTN pain participants that displayed pain intensity ratings in all four pain blocks were included in this post-hoc analysis. Group-level t-values of two-sample t-tests are also provided comparing connectivity change in each pain block of the fluctuating pain participants against the mean connectivity values across all evenly-spaced blocks in stable participants. *Ipsi = ipsilateral, PI = posterior insula, PCC = posterior cingulate cortex, dlPFC = dorsolateral prefrontal cortex, sgACC = subgenual anterior cingulate cortex.*

| **Region** | **Fluctuating (n=11)** | | | | **Stable (n=11)** | | | | | | | **F-value** | **p-value** |
| --- | --- | --- | --- | --- | --- | --- | --- | --- | --- | --- | --- | --- | --- |
| ***highest > lowest Pain*** | | |  | | |  |  | |  |  |  |  |  |
| Ipsi PI | 0.015 | -0.001 | 0.007 | 0.045 | 0.016 | | | 0.006 | | 0.011 | 0.016 | 6.682 | <0.001 |
| two-sample t-test p-value | 0.47 | 0.21 | 0.36 | 0.03 |  | | |  | |  |  |  |  |
| PCC | -0.006 | 0.003 | 0.014 | 0.025 | 0.002 | | | 0.004 | | 0.001 | 0.005 | 4.503 | 0.002 |
| two-sample t-test p-value | 0.11 | 0.40 | 0.18 | 0.008 |  | | |  | |  |  |  |  |
| Ipsi Hippocampus | -0.011 | 0.008 | 0.014 | 0.039 | 0.008 | | | 0.009 | | 0.021 | 0.013 | 5.611 | <0.001 |
| two-sample t-test p-value | 0.009 | 0.31 | 0.46 | 0.14 |  | | |  | |  |  |  |  |
| Ipsi dlPFC | -0.006 | -0.003 | -0.001 | 0.006 | 0.005 | | | 0.011 | | 0.006 | 0.008 | 2.272 | 0.023 |
| two-sample t-test p-value | 0.003 | 0.006 | 0.15 | 0.41 |  | | |  | |  |  |  |  |
| ***Highest < Lowest Pain*** | | |  | | |  |  | |  |  |  |  |  |
| sgACC | 0.020 | 0.005 | 0.009 | -0.003 | -0.003 | | | -0.005 | | -0.003 | -0.005 | 2.954 | 0.004 |
| two-sample t-test p-value | 0.002 | 0.03 | 0.02 | 0.29 |  | | |  | |  |  |  |  |
